# Effectiveness of traditional Chinese medicine formulas combined with acupuncture in the treatment of ovulation dysfunction infertility: A systematic review and meta-analysis

**DOI:** 10.1097/MD.0000000000034310

**Published:** 2023-07-07

**Authors:** Jingwen Mo, Yiyi Zhang, Ni Jin, Yi Zhou

**Affiliations:** a College of Basic Medicine, Chengdu University of Traditional, Chinese Medicine, Chengdu, Sichuan, China.

**Keywords:** acupuncture, infertility, meta-analysis, ovulatory disorders, randomized controlled trial, systematic evaluation, traditional Chinese medicine

## Abstract

To evaluate the efficacy and safety of traditional Chinese medicine formulas combined with acupuncture for the treatment of ovulation dysfunction infertility (ODI).

**Methods:**

From January 1, 2018 to March 12, 2023, 7 electronic databases, PubMed, EMBASE, Web of Science, Cochrane Library, CNKI, Wanfang Database, and CBM, were systematically searched to identify eligible randomized controlled trial studies.

**Results:**

Meta analysis showed that traditional Chinese medicine combined with acupuncture can more effectively improve sex hormone levels compared to Western medicine alone, including follicle stimulating hormone (FSH) in older patients (standardized mean difference [SMD]: 3.00; 95% confidence interval [CI]: 2.35–3.66; *P* = .024, I^2^ = 28%), FSH in younger patients (SMD: 0.45; 95% CI: −0.15, 1.05; *P* = .03, I^2^ = 71%), estradiol (E2) (SMD: 7.50; 95% CI: v0.47, 15.48; *P* < .00001, I^2^ = 99%), and progesterone (P) (SMD: 2.20; 95% CI: 2.07–2.33; *P* < .00001, I^2^ = 29%). Compared to Western medicine alone, traditional Chinese medicine combined with acupuncture also had a better effect to increase ovulation rate (risk ratio [RR]: 2.46; 95% CI: 1.72–3.52; *P* < .00001, I^2^ = 0%), pregnancy rate (RR: 2.50; 95% CI: 1.96–3.18; *P* < .00001, I^2^ = 0%), maximum follicle diameter (MFD) (SMD: 2.27; 95% CI: 1.37–3.16; *P* < .00001, I^2^ = 91%), and endometrial thickness (SMD: 1.71; 95% CI: 1.31–2.11; *P* < .00001, I^2^ = 87%). The combination of traditional Chinese medicine and acupuncture also had better effects on quality of life (RR: 0.19; 95% CI: 0.15–0.23; *P* < .00001, I^2^ = 0%) and reduced adverse reactions (RR: 0.15; 95% CI: 0.05–0.48; *P* = .001, I^2^ = 0%), compared to Western medicine alone.

**Conclusion:**

This study shows evidence that traditional Chinese medicine formulas combined with acupuncture are an effective and safe treatment approach. However, this conclusion requires further confirmation due to the insufficient quality of the included trials.

## 1. Introduction

Infertility refers to a low fertility state, which, in women, is defined as being unable to achieve a clinical pregnancy after 1 year while not on contraceptive treatment and maintaining a regular lifestyle. Infertility is a common gynecological condition with a complex etiology and has become a global medical and social problem. Ovulation disorder refers to the inability of follicles to develop or ovulate after maturation in women of reproductive age. Ovulation disorder may occur due to various reasons, resulting in clinical symptoms such as menstrual disorders and infertility. Indeed, ovulation disorder is one of the main causes of infertility.^[1]^ In modern medicine, it is believed that ovulation dysfunction infertility (ODI) is primarily caused by aberrant neuroendocrine regulation of the hypothalamic pituitary ovarian (H-P-O) axis.

Currently, modern medical research on ODI is mostly based on clinical symptoms and works in reverse to infer the genetic and molecular mechanisms of the endocrine axis to clarify treatment options. Treatment methods include oral ovulation promoting drugs,^[2]^ intramuscular injection of human chorionic gonadotropin,^[3]^ abdominal cavity drilling for ovulation, and assisted reproductive technology.^[4]^ However, there are significant controversies about the clinical efficacy of such treatments; for example, certain drugs are accompanied by a high rate of miscarriage, and many are expensive, require a lengthy treatment cycle, are limited by the development of drug resistance, and have a high incidence of adverse reactions. Therefore, simple Western medicine is not ideal for improving the overall state of the uterus and female reproductive system.

The guiding principles of traditional Chinese medicine, acupuncture and moxibustion follow the concept of syndrome differentiation and treatment. These practices focus on individualized treatment and have the advantages of low cost, high efficiency, high safety, and holistic treatment. From the perspective of traditional Chinese medicine, the etiology of ODI has a basis in kidney deficiency, commonly relating to liver depression, blood stasis, phlegm and dampness caused by Chong Ren imbalance.^[5]^ Due to the complex etiology and pathogenesis of ovulatory dysfunction, traditional Chinese medicine prescribed for this condition is often combined with acupuncture and moxibustion in clinical practice. However, the effectiveness and safety of such a combination treatment have not yet been systematically evaluated. Therefore, this study aims to evaluate the effectiveness and safety of Chinese medicine combined with acupuncture for the treatment of ODI by performing a meta-analysis.

## 2. Materials and Methods

### 2.1. Inclusion and exclusion criteria for randomized controlled trials

#### 2.1.1. Research object.

Patients with a definite diagnosis of ODI.

#### 2.1.2. Intervention measures.

The control group was treated with Western medicine, and the treatment group was treated with Chinese medicine combined with acupuncture, either in addition to the Western medicine prescribed to the control group or alone. The prescription types of TCM include decoction, pill and capsule.

#### 2.1.3. Outcome index.

Frequency of adverse events; efficacy rate; endometrial thickness; maximum follicle diameter (MFD); ovulation rate; pregnancy rate; sex hormone levels.

#### 2.1.4. Exclusion criteria.

The treatment group was treated with acupuncture alone or acupuncture combined with Western medicine. The treatment group was treated with ear or acupoint injection combined with traditional Chinese medicine. The control group was treated with traditional Chinese medicine or acupuncture. Reviews, abstracts, letters, conference papers, theses, case reports, case series reports, and animal experiments.

### 2.2. Literature retrieval strategy

Two authors searched 7 databases: PubMed, EMBASE, Web of Science, Cochrane Library, China National Knowledge Infrastructure, Wanfang Database, and Chinese BioMedical Literature Database, from January 1, 2018 to March 12, 2023. There were no language restrictions on publications. For studies with incomplete data, we contacted the authors to obtain relevant information. The search keywords were: “ovulation disorders,” “infertility,” “acupuncture,” “electroacupuncture,” “traditional Chinese medicine,” and “Chinese medicine.”

### 2.3. Literature screening and data extraction

Two reviewers (MJW and ZYY) independently screened the titles and abstracts of each record based on the inclusion criteria. For indistinguishable title/abstract records, the full texts were retrieved for further evaluation. Finally, any disagreement was resolved through discussion between the 2 reviewers or consultation with a third reviewer (JN). The following data were extracted: author, year of publication, number of patients, age, country，intervention measures, reference drugs, course of disease, outcome indicators, and intervention period.

The Cochrane Handbook (edition 5.1.0) and Review Manager software (edition 5.4.0) were used to assess the risk of bias in the included studies. The risk of bias included 7 aspects: randomization, allocation concealment, blinding of participants and personnel, blinding of outcome assessment, incomplete outcome data, selective outcome reporting, and other sources of bias. Specific risks of bias were used to divide studies into high-, low-, and unclear-risk groups. Finally, all studies that displayed selective outcome reporting and other sources of bias were considered at risk of ambiguity. Risk of bias assessment was conducted by 2 authors, and any differences were resolved through discussion between the 2 authors.

Review Manager software (edition 5.4.0) was used for quantitative synthesis. Discrete variables were analyzed using risk ratio (RR) and 95% confidence interval (CI). Continuous variables were assessed using the standardized mean difference (SMD) and 95% CI. Heterogeneity was estimated using Cochran Q test and the I^2^ statistic. When I^2^ < 50%, the fixed-effects model was adopted, and when 50% <I^2^ < 75%, the random-effects model was applied. I^2^ > 75% was considered to indicate high heterogeneity, and the source of heterogeneity was analyzed by establishing subgroups. The threshold for statistical significance was set at *P* < .05. A funnel plot, which included more than 10 studies, was used to evaluate the publication bias of the outcomes.

## 3. Results

### 3.1. Study inclusion and exclusion process

**Figure 1.**
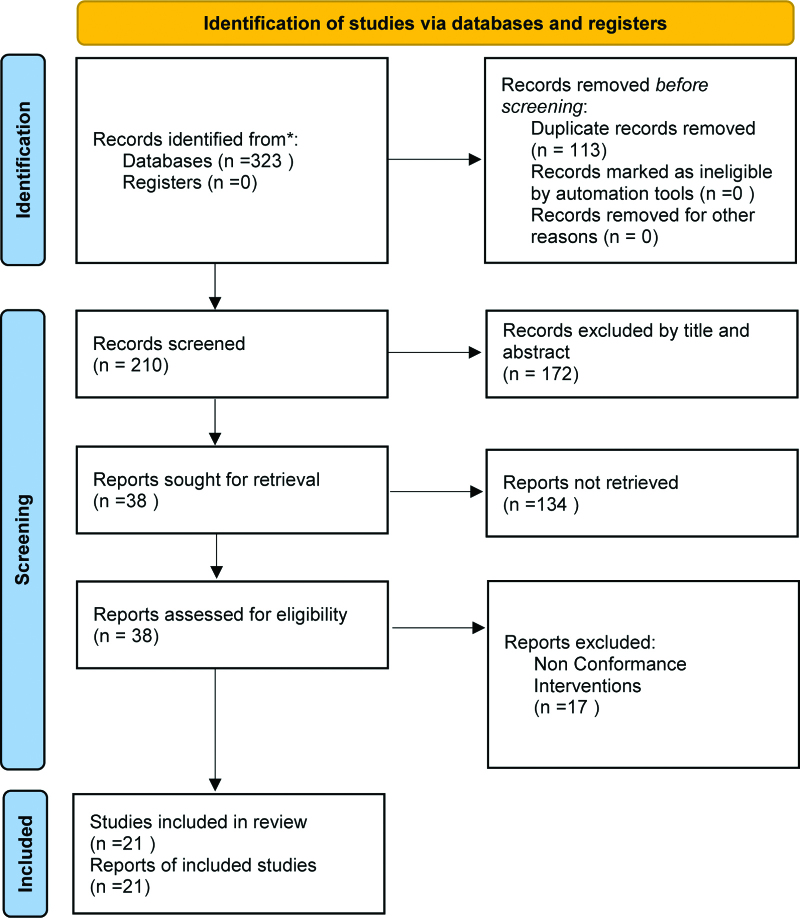
The PRISMA flowchart of the selection process. PRISMA = Preferred Reporting Items for Systematic Review and Meta-analysis.

A total of 323 studies were identified by searching the databases and 210 articles remained after removing duplicate literature. After layer-by-layer screening, 21 studies were included in the meta-analysis. A flowchart of the selection process is shown in Figure 1.

### 3.2. The basic characteristics of the included studies and the results of bias risk assessment

**Figure 2.**
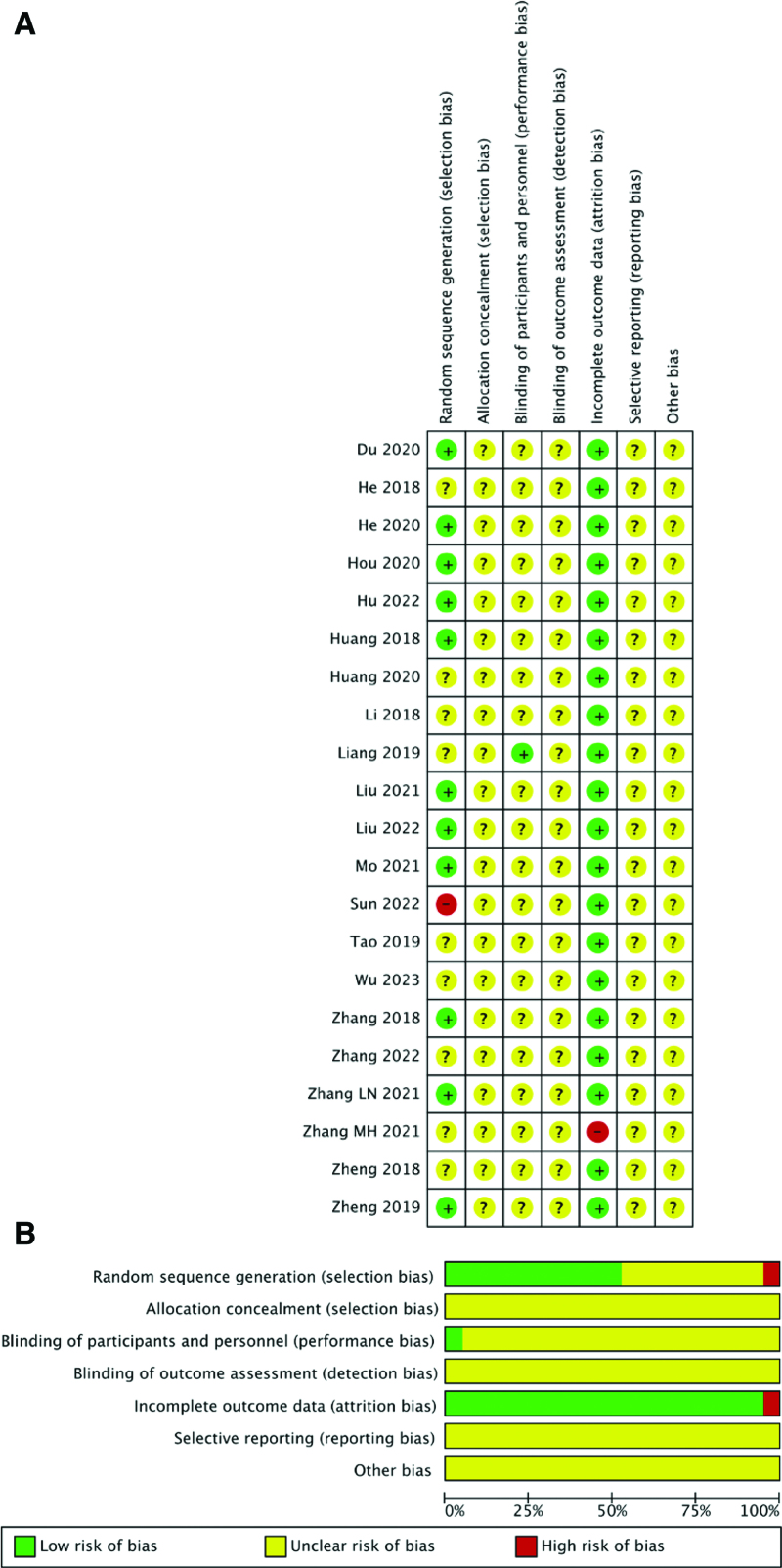
Summary of the risk of bias.

The general characteristics of the study population from 21 included studies^[6–26]^ are summarized in Tables 1 and 2. In brief, across the period from 2018 to 2023, 1841 patients were included, including 921 patients in the treatment group and 920 patients in the control group. The risk of bias for each study was assessed using the Cochrane risk-of-bias tool. A summary of the risk of bias is shown in Figure 2.

**Table 1:** Characteristics of included studies (1).

Study	Yr	Country	Age (yr) (mean ± SD)		Course of disease (yr) (mean ± SD)	
			T	C	T	C
Zheng et al	2018	China	28.48 ± 3.12	28.83 ± 3.35	2.07 ± 1.17	2.87 ± 2.27
Liu et al	2021	China	29.35 ± 7.07	30.01 ± 6.25	6.00 (1–11)	6.50 (1–12)
Zheng et al	2019	China	28.00 ± 3.00	29.00 ± 3.00	2.07 ± 1.17	2.87 ± 2.27
Mo et al	2021	China	32.13 ± 4.46	29.97 ± 5.10	——	——
He et al	2020	China	27.00 ± 5.00	25.00 ± 8.00	1.22 ± 0.27	1.27 ± 0.34
Wu et al	2023	China	30.44 ± 5.20	29.54 ± 4.70	2.59 ± 1.52	2.54 ± 1.60
Liu et al	2022	China	27.82 ± 3.76	28.35 ± 3.12	3.87 ± 1.23	4.12 ± 0.84
Huang	2020	China	32.08 ± 5.39	32.54 ± 5.48	——	——
Zhang	2018	China	31.80 ± 7.10	32.10 ± 6.80	2.80 ± 0.60	3.10 ± 0.80
Du et al	2020	China	31.31 ± 4.65	32.37 ± 4.83	3.33 ± 1.61	3.06 ± 1.55
Huang	2018	China	35.57 ± 2.51	34.61 ± 2.55	——	——
Li	2018	China	25.80 ± 2.56	24.80 ± 2.16	4.15 ± 1.20	4.35 ± 1.10
Hu	2022	China	30.48 ± 2.28	31.22 ± 2.24	4.30 ± 2.00	4.60 ± 1.50
Zhang	2022	China	26.97 ± 2.59	26.30 ± 2.28	4.10 ± 0.88	4.04 ± 0.86
Zhang MH	2021	China	26.70 ± 3.60	25.80 ± 2.70	3.79 ± 1.65	3.68 ± 1.34
Liang	2019	China	28.40 ± 2.60	28.10 ± 2.50	4.20 ± 1.10	4.30 ± 1.20
Tao	2019	China	28.4	27.8	——	——
Zhang LN	2021	China	30.02 ± 3.37	29.85 ± 2.98	3.12 ± 0.81	2.96 ± 0.74
Hou	2020	China	29.40 ± 4.80	29.40 ± 4.90	3.20 ± 1.80	4.00 ± 1.40
He et al	2018	China	26.2 ± 2.3	26.5 ± 2.1	1.6 ± 0.3	1.9 ± 0.5
Sun et al	2020	China	35.12 ± 2.38	35.28 ± 2.61	3.22 ± 0.57	3.18 ± 0.61

**Table 2:** Characteristics of included studies (2).

Study	Intervention		Number of patients (T/C)	Intervention period	Relevant outcomes
	T	C			
Zheng et al	AP+①	CC + HCG	60 (27/33)	3 mo	②
Liu et al	AP+①	LE	40 (20/20)	3 mo	②③④
Zheng et al	AP+①	CC + HCG	57 (27/30)	3 mo	②③⑤⑥⑧
Mo et al	AP+①	CC	60 (30/30)	6 mo	⑥
He et al	EA+①	LE + HCG	80 (40/40)	3 mo	③⑥
Wu et al	AP+①	CC	76 (38/38)	3 mo	⑤⑥⑦⑧
Liu et al	AP+①+CC	CC	80 (40/40)	9 mo	③⑥
Huang	AP+①	CC + HCG	80 (40/40)	6 mo	②③④
Zhang	AP+①	CC	94 (47/47)	3 mo	⑤⑥⑦
Du et al	AP+①+CC	CC	103 (51/52)	3 mo	②③④⑥
Huang	AP+①	CC + HCG	100 (50/50)	3 mo	②③⑤⑥
Li	AP+①	CC + HCG	140 (70/70)	3 mo	②③④
Hu	AP+①+CC	CC	55 (27/28)	6 mo	③⑤⑥
Zhang	AP+①	LE	90 (45/45)	3 mo	②⑦
Zhang MH	AP+①+CC	CC	80 (40/40)	1 mo	③④⑥⑦
Liang	AP+①	CC	90 (45/45)	0.5 mo	③⑤⑥⑧
Tao	AP+①+LE	LE	132 (72/60)	6 mo	⑤⑥
Zhang LN	AP+①+CC	CC	98 (49/49)	3 mo	②③④⑦
Hou	AP+①+CC	CC	110 (55/55)	3 mo	②④⑥⑦
He et al	AP+①	CC + HCG	120 (60/60)	3 mo	②③④⑥⑦
Sun et al	AP+①	CC	96 (48/48)	6 mo	②

AP = acupuncture, C = control group, CC = clomiphene citrate, EA = electroacupuncture, HCG = human chorionic gonadotropin, LE = letrozole, T = treatment group.

①Chinese medicine formulas; ②efficacy rate; ③endometrial thickness; ④maximum follicle diameter; ⑤ovulation rate; ⑥pregnancy rate; ⑦sex hormone levels; ⑧frequency of adverse events.

### 3.3. Meta-analysis results

#### 3.3.1. Efficacy rate.

**Figure 3.**
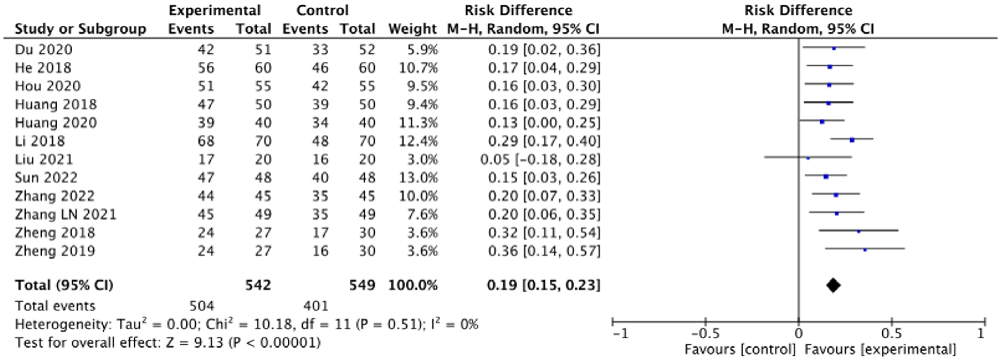
Forest plot of the efficacy rate comparing the experimental and control groups.

Clinical efficacy of the treatment was assessed based on pregnancy, menstrual improvement, and other symptoms. 12 studies reported efficacy rates, which were significantly higher in the experimental group than the control group (RR: 0.19; 95% CI: 0.15–0.23; *P* < .00001, I^2^ = 0%). The heterogeneity among the studies was very low. A forest plot of the efficacy rate is shown in Figure 3.

#### 3.3.2. Pregnancy rate.

**Figure 4.**
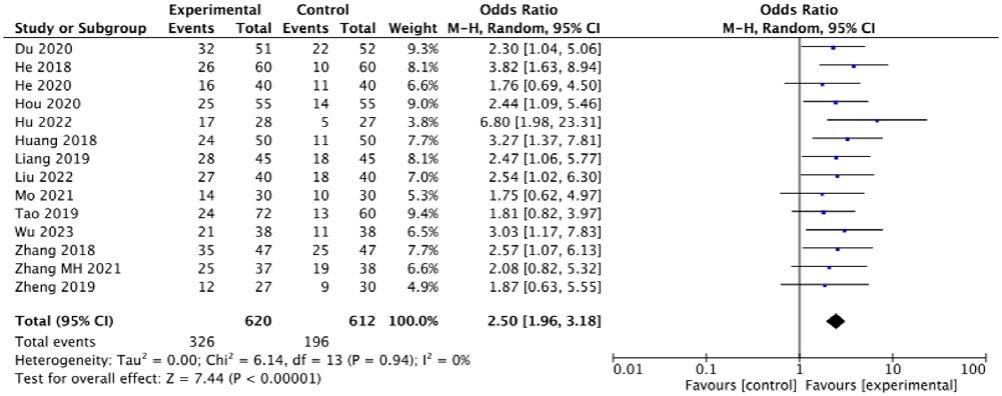
Forest plot of the pregnancy rate comparing the experimental and control groups.

Among the 21 included studies,14 reported the pregnancy rate. Comprehensive analysis showed that the pregnancy rate in the experimental group was significantly higher than that in the control group (RR: 2.50; 95% CI: 1.96–3.18; *P* < .00001, I^2^ = 0%). Again, the heterogeneity among the studies was extremely low. A forest plot of the pregnancy rate is shown in Figure 4.

#### 3.3.3. Ovulation rate.

**Figure 5.**
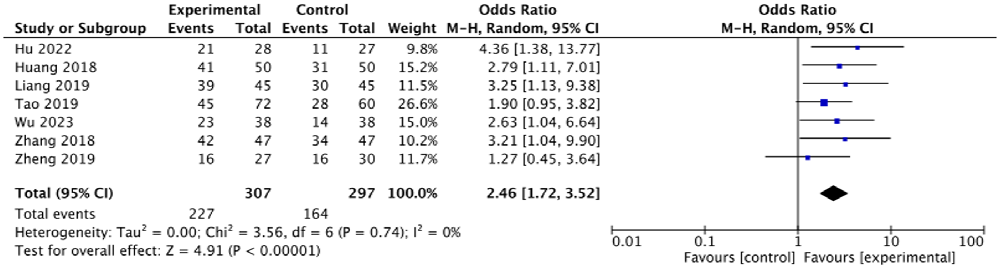
Forest plot of the ovulation rate comparing the experimental and control groups.

Seven studies reported ovulation rates, which showed that the ovulation rate in the experimental group was higher than that in the control group, with a statistically significant difference between the 2 groups (RR: 2.46; 95% CI: 1.72–3.52; *P* < .00001, I^2^ = 0%). There was very low heterogeneity among the studies. A forest plot of the ovulation rate is shown in Figure 5

#### 3.3.4. Maximum follicle diameter.

**Figure 6.**
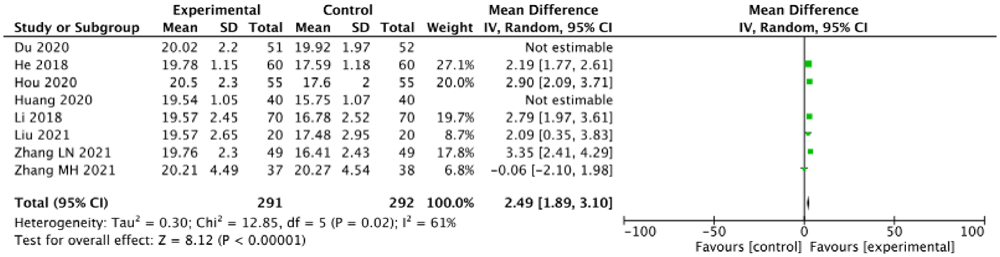
Forest plot for MFD comparing the experimental and control groups. MFD = maximum follicle diameter.

MFD was reported in 8 studies. MFD was significantly higher in the experimental group than the control group (SMD: 2.27; 95% CI: 1.37–3.16; *P* < .00001, I^2^ = 91%). However, there was a high degree of heterogeneity among the studies. After eliminating the Huang 2020 and Du 2020 studies, which were identified as sources of heterogeneity, the heterogeneity in the MFD decreased (SMD: 2.49; 95% CI: 1.89–3.10; *P* < .00001, I^2^ = 61%). The results suggested that Chinese medicine combined with acupuncture could effectively improve MFD. A forest plot of MFD is shown in Figure 6.

#### 3.3.5. Endometrial thickness.

**Figure 7.**
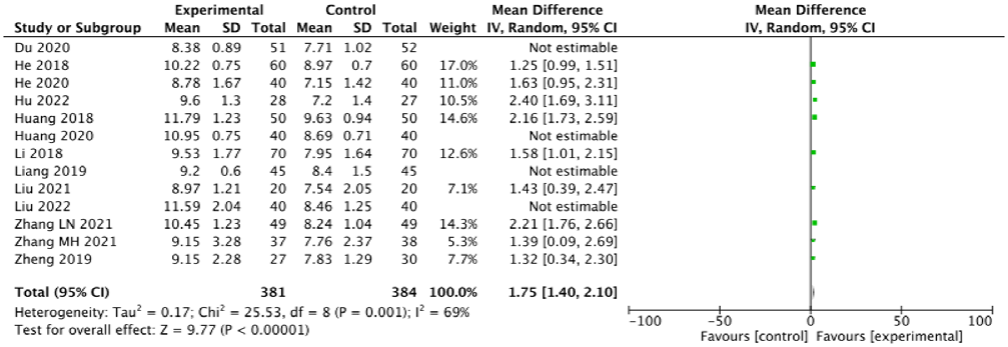
Forest plot for endometrial thickness comparing the experimental and control groups.

Endometrial thickness was reported in 13 studies. The results showed that endometrial thickness in the experimental group was significantly higher than that in the control group (SMD: 1.71; 95% CI: 1.31–2.11; *P* < .00001, I^2^ = 87%). However, the heterogeneity among different studies was very high. Four studies, Du 2020, Huang 2020, Liang 2019 and Liu 2022, were identified as likely sources of heterogeneity and excluded, after which the heterogeneity for endometrial thickness decreased to some extent (SMD: 1.75; 95% CI: 1.40–2.10; *P* < .00001, I^2^ = 69%). Hence, overall, our results suggest that Chinese medicine combined with acupuncture can effectively increase endometrial thickness. A forest plot of endometrial thickness is shown in Figure 7.

#### 3.3.6. Sex hormone levels (follicle stimulating hormone [FSH], estradiol [E_2_], P).

**Figure 8.**
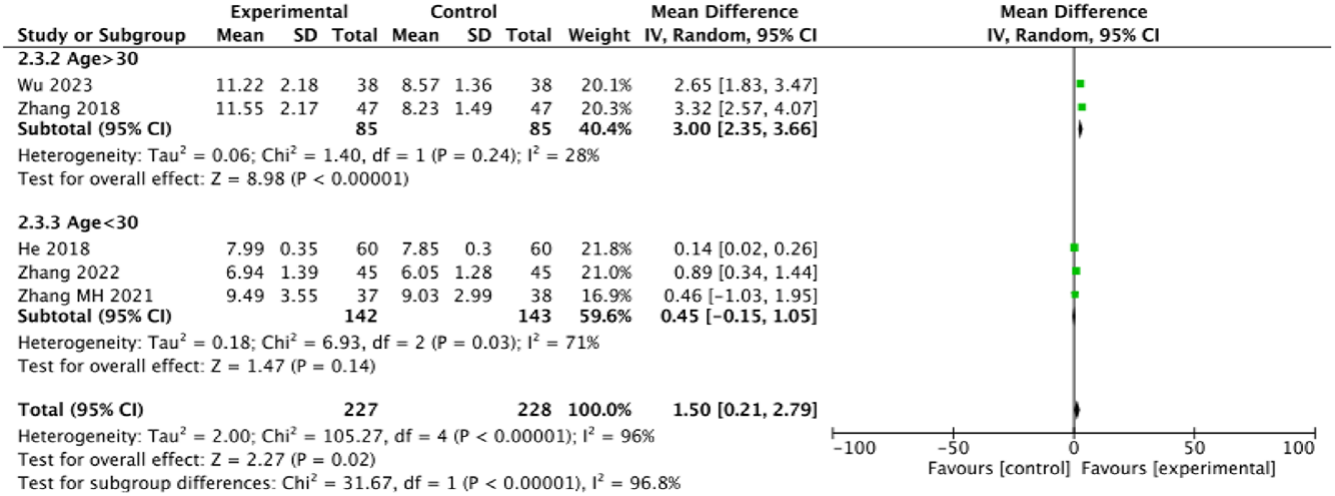
Forest plot for FSH comparing the experimental and control groups. FSH = follicle stimulating hormone.

**Figure 9.**
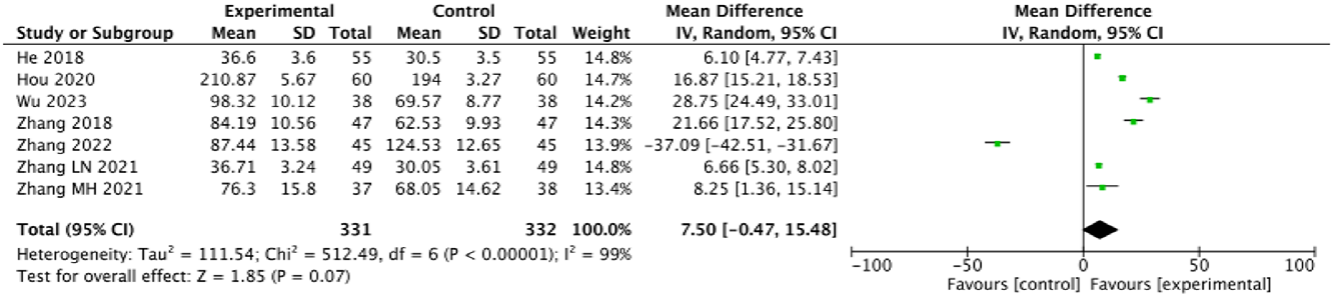
Forest plot for E_2_ comparing the experimental and control groups. E2 = estradiol.

**Figure 10.**
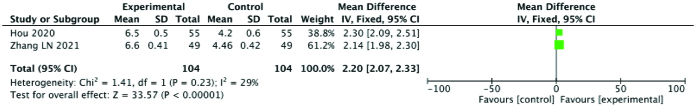
Forest plot for P comparing the experimental and control groups. P = progesterone.

Five studies examined blood levels of FSH. To analyze FSH levels, the patients were divided into 2 groups according to age. The results of meta-analysis showed that the FSH level of the experimental group was significantly higher than that of the control group (SMD: 3.00; 95% CI: 2.35–3.66; *P* = .024, I^2^ = 28%) for older patients and (SMD: 0.45; 95% CI: −0.15, 1.05; *P* = .03, I^2^ = 71%) for younger patients. Seven studies reported E_2_ levels, and meta-analysis suggested that E_2_ levels in the experimental group were also significantly higher than those in the control group (SMD: 7.50; 95% CI: −0.47, 15.48; *P* < .00001, I^2^ = 99%). However, after subgroup analysis based on disease course and course of treatment, the heterogeneity of each group remained high, and no source of heterogeneity was found, suggesting that these meta-analysis results should be treated with caution. Finally, 2 studies documented changes in progesterone (P) levels. Upon meta-analysis, the results showed that the P level of the experimental group was significantly higher than that of the control group (SMD: 2.20; 95% CI: 2.07–2.33; *P* < .00001, I^2^ = 29%). In general, the sex hormone levels of patients with ODI were higher among those who were treated with acupuncture combined with Chinese medicine compared to Western medicine alone. Therefore, it is likely that acupuncture combined with traditional Chinese medicine can regulate the endocrine system to increase the release of sex hormones. Forest plots of sex hormone levels are summarized in Figures 8, 9, and 10.

#### 3.3.7. Frequency of adverse events.

**Figure 11.**
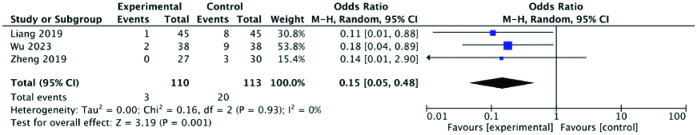
Forest plot of the frequency of adverse events comparing the experimental and control groups.

Adverse effects were mentioned in 3 studies. The meta-analysis results showed that the frequency of adverse reactions in the experimental group was far lower compared to the control group (RR: 0.15; 95% CI: 0.05–0.48; *P* = .001, I^2^ = 0%). The difference between the 2 groups was statistically significant and the heterogeneity was very low. A forest plot illustrating the frequency of adverse events is displayed in Figure 11.

#### 3.3.8. Funnel plot.

**Figure 12.**
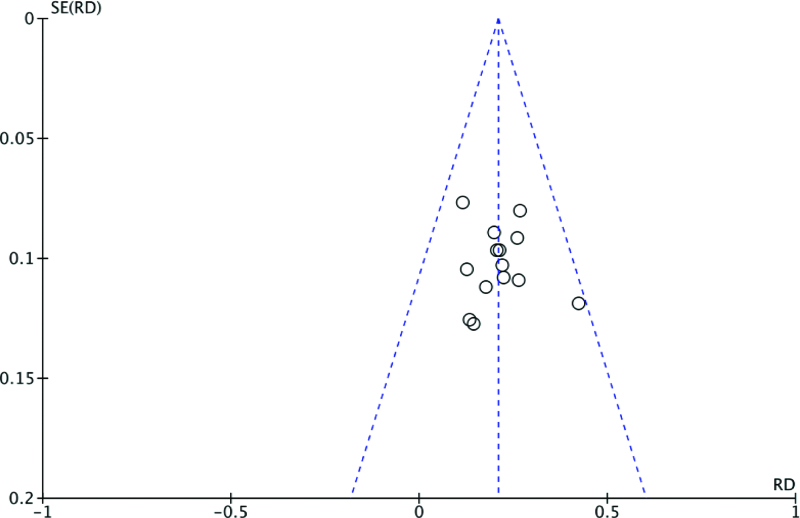
Funnel plot for publication bias of pregnancy rate.

The possibility of publication bias for pregnancy rate was evaluated using a funnel plot. The funnel plot distributions of outcomes were symmetrical. Funnel plots for the potential publication bias relating to pregnancy rate are shown in Figure 12.

## 4. Discussion

### 4.1. Principal findings

This study is the first to evaluate the efficacy and safety of traditional Chinese medicine (TCM) compounds combined with acupuncture for the treatment of ODI. The meta-analysis results suggest that, compared with Western medicine alone, TCM combined with acupuncture can improve the clinical efficacy, increase sex hormone levels, increase ovulation rate and pregnancy rate, increase MFD and endometrial thickness, improve patients’ clinical symptoms, and improve the success rate of conception. Moreover, safety was assessed by investigating the frequency of the adverse reactions, which were much less common in the group that received acupuncture combined with Chinese herbal medicine for the treatment of ODI compared to the control group.

### 4.2. Therapeutic mechanism

The core pathology of ODI is follicular dysplasia or excessive follicular wall hyperplasia, resulting in failure of the follicular wall to rupture and consequent follicular atresia,^[27]^ which is the main cause of primary infertility. At present, the main therapeutic approaches for ODI include oral ovulation-inducing drugs, intramuscular injection of HCG, and assisted reproductive technology, but these are limited by long treatment cycles and high cost. Therefore, there is an urgent need for novel therapeutic measures. In China, traditional Chinese medicine and acupuncture have been widely used for the treatment of ODI. Because TCM treatment is based on syndrome differentiation, good clinical effects have been achieved. Moreover, recent studies have explored and elucidated many of the mechanisms through which acupuncture and traditional Chinese medicine can achieve efficacy in the treatment of ODI.

#### 4.2.1. Mechanisms of TCM treatment.

Through the lens of traditional Chinese medicine, the normal reproductive function of women is closely related to the function of the kidney. For example, Professor Xia explained that the biggest cause of ODI is the lack of kidney Yin, which is insufficient to nourish the kidney essence, resulting in hampered development of the ovum.^[28]^ Professor Xia proposed the method of tonifying the kidney to regulate the reproductive cycle.^[28]^ Zhu et al^[29]^ studied the relationship between the H-P-O axis in the context of kidney deficiency and depression, and believed that traditional Chinese medicine could be used to increase the levels of sex hormones, including FSH, luteinizing hormone and E_2_, thus improving the function of the H-P-O axis. In addition, Zhu et al also found that traditional Chinese medicine can impact the oxidative stress response, as observed through decreased levels of reactive oxygen species, malondialdehyde, and glutathione peroxidase, and increased superoxide dismutase, advanced oxidation protein products, and other oxidative stress-related indexes.

Similarly, Professor Fang^[30]^ also reported that tonifying the kidney and promoting blood circulation can improve ovarian function and optimize the uterine environment by regulating the H-P-O axis, thus promoting the formation of high-quality embryos. In line with this, animal experiments^[31]^ found that Bushen Huoluofang can promote follicle development and increase the number of follicles by modulating the expression of IGFBP mRNA in the ovaries of rats, to achieve an overall effect of promoting ovulation. Furthermore, another TCM compound^[32]^ has been shown to promote the release of gonadotropin releasing hormone (GnRH), enhance the endocrine function of the pituitary gland, increase circulating levels of GnRH, improve the endocrine function of the ovary, and promote the development and excretion of follicles.

#### 4.2.2. Mechanisms of acupuncture therapy.

Acupuncture treatment of ODI has gradually become a research hotspot. Huang et al^[33]^ believed that the mechanism of acupuncture to promote ovulation may involve improving the levels of sex hormones in patients by regulating hypothalamic function and promoting the release of pituitary neurotransmitters, thus regulating ovarian function to restore ovulation. Indeed, other studies^[34–36]^ have shown that acupuncture can regulate the function of the H-P-O axis by activating the brain dopamine system. Ai et al^[37]^ found that acupuncture could promote dopaminergic activity in the brains of ODI patients, improve sex hormone levels, and increase the clinical pregnancy rate. Furthermore, the practice of electroacupuncture to induce ovulation has been associated with circulating levels of beta-endorphin immunoactive substances and pituitary gonadotropin secretion.^[38]^ In animals,^[39]^ central opioid peptides, especially β-endorphin, can act as inhibitors of GnRH. Therefore, acupuncture, which can affect the activity of the opioid peptide system, is likely to regulate the H-P-O axis through changes in β-endorphin and other opioid peptides.

### 4.3. Chinese medicine formulas and acupoint

Traditional Chinese medicine doctors have proposed a special ODI treatment method based on the corresponding theory of heaven and man, which simulates normal menstrual cycle changes and periodically gives different traditional Chinese medicine. It is called artificial cycle administration.

The focus of menstrual treatment is on promoting blood circulation and regulating menstruation, with the addition or subtraction of Wuwei Tiaojing Tang for treatment; The focus of early treatment after menstruation is on nourishing yin and blood, as well as using yin to support yin, with the addition and subtraction of Erzhi Pills for treatment; The focus of treatment in the mid-term after menstruation is to nourish yin and blood, with the treatment of Jianpi Ziyin Tang; The focus of treatment in the late stage of menstruation is to nourish yin and assist yang, with the addition or subtraction of Wuzi zhongyu Decoction for treatment; The focus of ovulation treatment during menstruation is to tonify the kidney and promote blood circulation, with the treatment of tonifying the kidney and promoting ovulation formula; The focus of treatment in the first half of the menstrual cycle is to tonify the kidney and assist yang, and the patient is treated with Yulin Zhu; The focus of treatment in the first and second half of the menstrual cycle is to fill the Chongren Blood Sea, and treat it with Jianpi Wenshen Tang.^[40–43]^

The Chinese traditional medicine has a long history of using acupuncture. In the treatment of ODI, doctors artificially stimulate the body with acupuncture to regulate itself and restore normal ovulation. Through organizing and reading literature, it was found that the traditional acupuncture treatment plans mainly selected Guanyuan (CV4), Sanyinjiao (SP6), and Zigong (EX-CA1).Then, according to the different systems of the patients, other acupoints are matched. For example, for patients with kidney deficiency, select the Shenshu (BL23), for patients with liver depression, select the Ganshu (BL18), and for patients with phlegm dampness internal obstruction, select the Pishu (BL20) and Fenglong (ST40).^[44–47]^

### 4.4. Implications for clinical practice and further research

Overall, many studies, including the present one, have shown that acupuncture combined with Chinese herbal medicine can exert a good effect on ODI. However, it is necessary to further study and screen the effective acupuncture points to form a standardized acupuncture program. High-quality, large-scale, multicenter clinical randomized controlled studies are needed to obtain more accurate analytical results. To produce studies with optimal quality, further research should prioritize careful unitization, allocation concealment, and blinding, clarify the inclusion and exclusion criteria, and establish consistent disease treatment standards.

### 4.5. Advantages of this study

The current systematic review focuses on the treatment of ODI with Western medicine alone or traditional Chinese medicine combined with acupuncture. To our knowledge, this study is the first systematic review and meta-analysis to discuss the effectiveness of acupuncture combined with traditional Chinese medicine in the treatment of ODI. This study provided some clarity on the efficacy of acupuncture combined with traditional Chinese medicine for the treatment of ODI. We focused on the effects of common forms of acupuncture rather than electroacupuncture, which reduced the chances of variability within the results arising from diverse forms of acupuncture. This enabled a more accurate assessment of the role of acupuncture in combination with Chinese herbal compounds for ODI.

### 4.6. Limitations

The limitations of this study are as follows. First, the level of evidence in this study is limited due to the lack of high-quality, multicenter, randomized controlled clinical studies. Second, some of the results showed great heterogeneity. Although subgroup analysis and one-by-one exclusion methods were used, in some cases the source of heterogeneity was still unable to be identified. This may be due to the complex effective ingredients and different formulations of traditional Chinese medicines. Moreover, since the included studies were all conducted in China, it is difficult to evaluate the efficacy and safety of acupuncture combined with acupuncture in different ethnic groups and regions.

## 5. Conclusion

This study shows that acupuncture combined with traditional Chinese medicine is a potential treatment method for ODI that can not only improve the efficacy rate, pregnancy rate, ovulation rate, MFD and endometrial thickness, but also enhance sex hormone levels, relieve patients’ clinical symptoms, improve the quality of follicle development, and improve the pregnancy environment. Although these conclusions require further validation due to the limitations of existing studies, the current evidence provides hope for further exploration.

## Author contributions

**Conceptualization:** Yiyi Zhang, Yi Zhou.

**Data curation:** Jingwen Mo.

**Formal analysis:** Jingwen Mo.

**Investigation:** Jingwen Mo.

**Methodology:** Jingwen Mo.

**Resources:** Jingwen Mo.

**Software:** Jingwen Mo.

**Supervision:** Yiyi Zhang, Ni Jin.

**Validation:** Yiyi Zhang, Ni Jin.

**Writing – original draft:** Jingwen Mo.

**Writing – review & editing:** Jingwen Mo.
